# Prophylactic Cranial Irradiation for Extensive-Stage Small-Cell Lung Cancer: A Controversial Area

**DOI:** 10.3389/fonc.2022.772282

**Published:** 2022-02-07

**Authors:** Shuyu Xue, Hanqiao Zeng, Shu Yan, Qianmeng Wang, Xiaojing Jia

**Affiliations:** Department of Tumor Radiotherapy, The Second Hospital of Jilin University, Changchun, China

**Keywords:** small-cell lung cancer, brain metastasis, extensive disease, prophylactic cranial irradiation, radiotherapy

## Abstract

Small-cell lung cancer (SCLC) is a highly aggressive malignant tumor that is prone to lead to the development of brain metastases (BM). The application of prophylactic cranial irradiation (PCI) has been regarded as an important technological advance made in cancer therapy to reduce the occurrence of BM and improve patient survival. The benefits of PCI in the treatment of limited-stage SCLC have been confirmed. However, there has been continuous controversy about the indications and advantages of PCI for extensive-stage SCLC (ES-SCLC) because of the conflicting results from two prospective trials. In this review, we aimed to discuss the relevant controversy and progress made in the clinical application of PCI in ES-SCLC.

## Introduction

Small-cell lung cancer (SCLC) accounts for only 13% of all newly diagnosed lung cancers (1) and has distinct pathological, clinical, and molecular characteristics from those of non-small-cell lung cancer (NSCLC). Despite relatively low incidence, SCLC is associated with a poor clinical prognosis due to its high metastatic potential.

Most patients with SCLC present with extensive disease (ED) and have a poor median overall survival (OS) without treatment (range: 2–4 months) (2). A total of 50% of patients with SCLC are at risk of developing brain metastases (BM) (3), and this group of patients only has a median OS of 4–5 months (4). At least 18% of patients with SCLC are first diagnosed with BM; this rate could increase to 25% with the use of more thorough tests, such as magnetic resonance imaging (MRI). In addition, as the disease progresses, the incidence of BM increases, with 80% of the patients developing BM within 2 years of diagnosis (5). The progression of BM is often accompanied by the appearance of neurological symptoms and impairment of health-related quality of life (QoL), suggesting that the median survival in patients is less than 6 months.

Since the 1980s, SCLC has been treated with radiotherapy and chemotherapy (6). Based on the high sensitivity to the combination treatment, up to 25% of patients gain lasting benefits (6). However, 30%–50% patients experienced relapse in the central nervous system (CNS) at later stages (6, 7). Systemic therapy is not effective at preventing BM because most anticancer drugs have poor ability to penetrate the blood–brain barrier. Therefore, reducing the incidence of BM has become an important challenge to improve patient survival. Many recent studies have focused on the treatment of BM because of its high propensity in SCLC (8).

While whole-brain radiation therapy (WBRT) has long been the standard treatment for limited and solitary BM in SCLC, the introduction of prophylactic cranial irradiation (PCI) in 1997 for the treatment of BM was a major breakthrough (9). A meta-analysis published in 1999, which included 987 patients with SCLC, used PCI as the standard treatment for those who responded to initial systematic treatment (7). The arm that received PCI had an absolute increase of 5.4% in the 3-year OS and a significant reduction in the 3-year incidence of BM (from 58.6% to 33.3%). Although most patients had limited disease (LD) and ED patients represented only 15% of the study population, similar benefits were observed in both subgroups.

Unlike the widespread use of PCI in patients with LD, the use of PCI in extensive-stage SCLC (ES-SCLC) has become controversial after the publication of two prospective studies with opposing conclusions (10, 11). Slotman et al. showed that PCI reduced the incidence of symptomatic BM and significantly improved progression-free survival (PFS) and OS in the randomized trial that included 286 ES-SCLC patients (10). Their study supported the benefits of PCI in the treatment of ES-SCLC patients (10). However, this study did not routinely perform brain imaging in the enrolled patients. Therefore, with advances in diagnostic imaging technology, increasing number of researchers have started questioning the survival benefit derived from this study. In an effort to validate this study, Takahashi et al. conducted a new randomized phase III trial and required enrolled patients to undergo MRI to confirm the absence of BM (11). In contrast to the findings of Slotman et al. that new trial was terminated early because no possible survival benefit from PCI was observed. Because of these contrasting results (**Table 1**), the use of PCI in the treatment of ES-SCLC has become a long-standing debate. Furthermore, with the improvement of imaging examination methods, the application of stereotactic body radiation therapy (SBRT), and the promotion of TNM staging in the treatment of SCLC (12), the scope and value of PCI in ES-SCLC treatment need to be urgently addressed. This article reviewed the main arguments and evidence concerning the PCI efficacy and potential toxicity to provide a clear perspective for the follow-up research and clinical application of PCI.

**Table 1:** Summary of EORTC and JCOG Trials.

Trial	Treatment	N	PFS, months (median)	OS, months (median)	1-year OS (%)	2-year OS (%)	Second-line chemotherapy administered, %
EORTC, 2007 [Slotman (10)]	PCI	143	3.5	6.7 (*P* = 0.003)	27	–	Not reported
	Observation	143	2.8	5.4	13	–	Not reported
JCOG, 2017 [Takahashi (11)]	PCI	113	2.3	11.6 (*P* = 0.094)	48.4	15.0	88
	Observation	111	2.4	13.7	53.6	18.8	89

EORTC, European Organization for Research and Treatment of Cancer; JOCG, Japan Clinical Oncology Group; PCI, prophylactic cranial irradiation; PFS, progression-free survival; OS, overall survival.

## PCI Treatment in Patients With ES-SCLC

Since the development of a combination of radiotherapy and chemotherapy for SCLC in the 1980s, CNS recurrence has occurred in 30%–50% of patients after brief remission, which often led to treatment failure (6, 7). Researchers’ experience with pediatric acute leukemia led to the introduction of PCI as treatment of SCLC. While reducing the incidence of CNS metastases may prevent CNS complications, clinical trials are required to demonstrate improved OS or improved QoL to promote the application of PCI in clinical practice.

### Meta-Analyses

In 1999, a meta-analysis by Auperin et al. implemented PCI as part of the treatment for SCLC patients with complete remission after initial treatment (7). Based on the analysis of seven randomized trials conducted between 1977 and 1994 that included 987 patients, they found that OS in patients who received PCI was improved compared with that in the observation group, with a relative risk of 0.84 (*P* = 0.01). PCI also reduced the cumulative 3-year incidence of BM (58.6% in the observation group vs. 33.3% in the treatment group, relative risk: 0.46). Moreover, the 3-year survival rate increased from 15.3% in the observation group to 20.7% in the treatment group, with an absolute increase of 5.4%. In addition, PCI had no effect on metastases at other sites and locoregional relapse; thus, the study concluded that PCI reduced the incidence of BM, which resulted in the improved OS. ED patients represented only 15% of the study population; however, similar benefits were observed in both subgroups. It should be noted that the enrollment conditions, treatment regimens, and evaluation criteria for patients in the analyzed studies varied widely. Furthermore, most trials included in this analysis evaluated the complete response (CR) using chest radiographs rather than CT scans.

Another meta-analysis of two randomized trials conducted in the 1980s by Arriagada et al. (13) yielded results similar to those shown by Auperin et al. (7). The risk of no CNS metastases or other recurrences within 5 years was 11% in the PCI group and 17% in the non-PCI group.

Notably, in these two meta-analyses, no patients were evaluated for CNS involvement using MRI, and some patients were from the pre-CT era. MRI has shown irreplaceable advantages in the monitoring of CNS diseases (14). In a study that included 481 patients from the Netherlands, the rate of BM diagnosed in the MRI era was 24%, compared with 10% in the CT era. Meanwhile, all patients diagnosed with BM in the CT era had related symptoms, whereas about half of those detected by MRI were asymptomatic (14). Therefore, the benefits of PCI reported by these two studies may have been due to the treatment of pre-existing BM.

### Prospective Studies

In 2007, Slotman et al. (10) from the European Organization for Research and Treatment of Cancer (EORTC) conducted a randomized trial of patients with ES-SCLC. Their results, together with those of previous meta-analyses, reinforced the role of PCI in ES-SCLC treatment. In this study, 286 patients who responded to systemic chemotherapy were enrolled and randomly assigned to the PCI or observation groups. The primary endpoint was the time to develop symptomatic BM, whereas the secondary endpoints were survival, QoL, toxic effects, and treatment costs. CT or MRI was performed when patients presented with symptoms of BM. There was no uniform dose/schedule for PCI, and the bioequivalent dose ranged from 25 to 39 Gy. They found that the incidence of symptomatic BM reduced by PCI, and the 1-year cumulative risk reduced from 40.4% in the observation arm to 14.6% in the PCI arm. There was an improvement in 1-year OS [27% in PCI arm vs. 13% in observation arm, hazard ratio (HR) = 0.68, *P* = 0.003] and median OS (6.7 months in PCI arm vs. 5.4 months in observation arm, *P* = 0.003). Meanwhile, median PFS was extended from 12 to 14.7 months in the PCI arm (*P* = 0.02). Based on the outstanding findings of this study, PCI has been recognized as an important therapeutic breakthrough and has been included in multiple guidelines (15, 16).

Over time, however, significant concerns have been raised regarding the general applicability of the EORTC trial results. First, the regimens for systemic chemotherapy were not strictly defined. Neither the response to chemotherapy was defined by the standard criteria (i.e., RECIST) nor the type of chemotherapy used. Second, the irradiation dose and classification method of enrolled patients were not uniform (i.e., 20 Gy was given in 5 fractions and 30 Gy in 10 fractions). Most importantly, brain imaging was neither mandatory prior to enrollment nor as a routine during follow-up (unless patients were symptomatic, suggesting BM). However, the incidence of BM in SCLC at diagnosis was as high at 25% when CNS examination was performed along with MRI, and a significant number of patients developed BM after first-line treatment and before PCI (14, 17). Thus, the failure of the EORTC trial to systematically screen enrolled patients for BM may have recommended PCI to some patients who already had BM, indicating that the improvement in OS may have been due to the treatment of the existing disease rather than a real preventive effect.

With the development of medical imaging technologies, MRI has gradually replaced CT as the main detection method for BM in SCLC patients. Seute et al. (14) reported that the use of MRI reduced the scope of application of PCI. Moreover, the detection rate of SCLC BM increased from 10% to 24%, and that of asymptomatic BM increased from 0% to 13%. Manapov et al. (17) reported that intracranial MRI examination was performed in patients with limited SCLC whose curative effect was evaluated as CR before PCI, of whom 32.5% of the patients had BM. Considering that MRI could distinguish some subclinical lesions that CT could not, the clinical value of PCI may have been overestimated.

To address the problems in the EORCT trial, Takahashi et al. (11) from the Japan Clinical Oncology Group (JCOG) conducted a new randomized phase III trial that enrolled patients with ES-SCLC. Unlike the EORTC trial, this new trial required patients to confirm the absence of BM using gadolinium-enhanced MRI after completing at least two cycles of initial platinum-based doublet chemotherapy. The inclusion criteria of the trial required any response of patients to systemic chemotherapy and no evidence of BM detected by MRI at 4 weeks after enrollment. A total of 224 patients in both experimental groups were required to undergo brain MRI every 3 months after enrollment for 1 year and at 18 and 24 months regardless of whether neurological symptoms appeared. Because of this, patients in the observation arm underwent regular active monitoring, whereas those in the PCI arm received PCI with a total dose of 25 Gy, which was divided into 10 fractions. The primary endpoint of this trial was OS, whereas the secondary endpoint was the time to BM development. Due to PCI ineffectiveness (the probability that PCI would improve OS was 0.011% compared with MRI monitoring alone), the study was terminated prematurely when it came to the interim analysis, with the results published in 2017. Median OS was 11.6 months [95% confidence interval (CI): 9.5–13.3] in the PCI arm and 13.7 months (95% CI: 10.2–16.4) in the observation arm, and HR was 1.27 (95% CI: 0.96–1.68, *P* = 0.094). The cumulative rates of BM in PCI group at 6, 12, and 18 months were 15.0% (95% CI: 9.2–22.3), 32.9% (24.3–41.7), and 40.1% (31.0–49.1), respectively, and 46.2% (36.7–55.2), 59.0% (49.1–67.6), 63.8% (54.072.1) in the observation group, respectively (*P* < 0.0001). This trial concluded that PCI was controversial to use for ES-SCLC patients who responded to initial chemotherapy and were confirmed to be free of BM. However, those who did not undergo PCI needed to receive regular monitoring by MRI during follow-up. Therefore, in patients with ES-SCLC, PCI should be replaced by active surveillance and salvage conformation/stereotactic brain radiotherapy once BM occurs.

The EORTC study (10) was the first randomized trial and became cornerstone in the field of PCI research. The Japanese trial reported that when PCI was replaced with regular MRI monitoring, patients receiving effective treatment after BM detection showed no decrease in OS. PCI has become more controversial as the JCOG trial (11) was published confirming the uncertainty of OS benefits of PCI. Conflicting data eventually led to amendments in the recommendations of the evidence-based clinical guidelines for patients with ES-SCLC (18).

The absence of mandatory MRI monitoring in the EORTC trial has questioned whether the improvement in OS was due to the treatment of existing BM rather than their prevention. Yin et al. conducted a meta-analysis of SCLC patients grouped according to whether brain imaging was mandatory (19). They found that PCI was associated with a statistically significant OS benefit when brain imaging was not mandatory (HR: 0.81, 95% CI: 0.67–0.99); however, whenever brain imaging was required, no OS benefit was noted (HR: 0.94, 95% CI: 0.74–1.18) (19). However, Hochstenbag et al. (20) reported that the proportion of asymptomatic BM patients detected by MRI during follow-up was approximately 15%, which we believe was insufficient to cause the observed OS benefit.

Information on the differences in the initial staging strategy is vital for understanding and applying the data reported in these studies. PCI was applied to two entirely different populations in the two studies: baseline brain imaging regardless of symptoms in the JCOG trial and symptom-directed imaging in the EORTC trial. The routine follow-up using brain MRI every 3 months in the JCOG trial made a considerable socio-economic impact, which would be difficult to achieve in daily clinical practice in many less developed countries. In addition, the 1-year OS in patients enrolled in JCOG (11) was significantly better than that in patients included in EORTC trial that could be observed in both PCI and control arms (10) (53.6% vs. 13.3% in the non-PCI group and 48.4% vs. 27.1% in the PCI group). Zhou et al. explained that some bias may have been due to biological heterogeneity between Asian and Caucasian populations (21). For example, studies in the JCOG randomized phase III trial that showed the effectiveness of irinotecan in improving OS in limited-stage SCLC (LS-SCLC) could not be confirmed in a similar North American study (22, 23). However, this may also suggest a selection bias. Indeed, a higher proportion of patients received second-line chemotherapy in JCOG (11) than in the EORTC (10) trial. In the JCOG trial (11), 40 patients (36%) in the control group and 29 (26%) in the PCI group received four-line chemotherapy. This was applied relatively rarely in patients with SCLC, and one might argue that it was not common in the clinical practice.

The median survival in patients with symptomatic BM is only 4–6 months (24–26). The EORTC and JCOG trials (10, 11) showed that PCI could significantly reduce the incidence of BM by two- to three-fold. In the JCOG trial (11), there was a higher cumulative rate of BM at all time points in the observation group, and ultimately 83% of patients (46% in the PCI group) required cranial radiotherapy. Similar to the EORTC trial, 59% of symptomatic BM patients in the non-PCI cohort received cranial radiotherapy compared with 8.3% in the PCI cohort (10). In addition, Nakamura et al. reported that among BM patients, those who underwent PCI had a better outcome (3-year OS, 17% vs. 0%, *P* = 0.005) and fewer BM incidents (>5 BM, 12% vs. 68%, *P* < 0.001), which suggests the possibility of performing salvage stereotactic radiosurgery (SRS) (27).The results of the retrospective studies were consistent with these findings. Chen et al. (28) found a significant increase in BM-free survival in the PCI group (*P* = 0.002), with a 1-year BM incidence of 17% in the PCI and 56% in the non-PCI groups. The Ontario study (29) also pointed out that PCI could prolong the median time to the incidence of BM (23.8 months in the PCI arm vs. 10.2 months in control arm). BM after PCI treatment can also be treated using salvage WBRT. Bernhardt et al. (30) pointed out that cranial re-irradiation with WBRT or SRS could be available for relieving symptoms with mild toxicity. In this case, SRS was no worse than WBRT and resulted in a better median survival (30). Suzuki et al. conducted a retrospective analysis of different treatments for BM occurrence/recurrence after PCI or WBRT and found similar results. Patients who received repeated WBRT treatment had a worse prognosis than those who received chemotherapy only, and patients who received SRS treatment showed the most ideal OS (31).

### Retrospective Studies

The current study results showed that MRI had a higher detection rate for subclinical lesions than CT, which could reduce the incidence of BM in patients with ES-SCLC; however, whether PCI can improve survival remains inconclusive. As conflicting prospective trials have carried considerable controversy to the field, PCI indication for ED-SCLC has quickly become a research hot spot. Independent letters, reviews, and meta-analyses by several research groups almost unequivocally affirm the survival benefits of PCI and oppose some of the recent changes in the guideline (32–34).

In 2016, Bernhardt et al. (35) conducted a retrospective study. This study analyzed the efficacy of PCI in 136 patients with ES-SCLC who responded to chemotherapy, and all patients underwent enhanced CT or MRI before PCI and BM was excluded. The median OS after PCI was 12 months, which was twice as long as that in the PCI group in the EORTC trial. The median OS in patients with MRI before PCI was not prolonged compared with that in patients who underwent enhanced CT (12 vs. 13 months, *P* = 0.200) (35). The median OS in the enrolled patients was significantly higher than that in patients enrolled in the EORTC and Japanese studies. This was considered to be related to the better general condition of the enrolled patients and the inevitable selection bias in retrospective studies.

There are additional four retrospective studies (28, 29, 36, 37) favoring the use of PCI to improve OS and intracranial control. Brain screening was performed in all studies, and MRI was the preferred imaging method, except a few cases wherein CT scans were used because of MRI contraindications (pacemaker, artificial implants, etc.). The median follow-up in these four studies was 23 months (range: 9–36 months).

The largest retrospective study based on the US National Cancer Database was performed by Sharma et al. in 2018 (36). Of the 4,257 patients with SCLC metastasis who were evaluated, 3,784 did not receive PCI and 473 received it. ES-SCLC patients enrolled in the study received chemotherapy and had no BM. The study matched propensity scores to factors related to PCI acceptance and OS, and the results showed an advantage for PCI even after excluding patients with survival of less than 6 or 9 months. PCI improved median survival (13.9 vs. 11.1 months, *P* < 0.001), 1-year survival probability (61.2% vs. 44%, *P* < 0.001), and 2-year survival probability (19.8% vs. 11.5%, *P* < 0.001).

In addition, Chen et al. (28) analyzed 204 ES-SCLC patients, among whom 45 (22.1%) underwent PCI, whereas the remaining 159 (77.9%) were in the observation group. Mandatory brain imaging tests were performed before the treatment to exclude BM. Patient response after 4–6 cycles of chemotherapy was evaluated. The median OS was significantly improved by the use of PCI (16.5 vs. 12.6 months [HR: 0.63; 95% CI: 0.41–0.96; *P* = 0.033]). In addition, PCI had a lower risk of BM (HR: 0.48; 95% CI: 0.30–0.76; *P* = 0.001), with a 1-year incidence of 17.1% and 55.9% in the PCI and observation groups, respectively. Multivariate analysis results showed that PCI was a good independent predictor of OS.

Bang et al. (29) analyzed 155 ES-SCLC patients without baseline BM in 2018. They found a statistically significant difference in OS (HR: 0.55; 95% CI: 0.39–0.77; *P* = 0.0005) and time to BM (HR: 0.40; 95% CI: 0.23–0.66; *P* = 0.0004) depending on the application of PCI. The median survival was 13.5 months in the PCI group versus 8.5 months in the observation group. Furthermore, the PCI group had a significantly increased 1- and 2-year OS (HR: 0.41; 95% CI: 0.29–0.57; *P* < 0.0001). The median time to develop BM was longer in the PCI group than in the observation group (23.8 vs. 10.2 months) (HR: 0.36; 95% CI: 0.21–0.60; *P* < 0.0001). There was significant difference in survival among patients who underwent PCI, regardless of whether they underwent brain imaging after chemotherapy.

Nicholls et al. (37) retrospectively analyzed 129 patients with ES-SCLC treated between 2008 and 2013. Of these, 13% received PCI and had a median OS of 13.6 months versus 5.6 months in those who did not receive PCI (*P* < 0.001).

Ge et al. published a meta-analysis that was criticized because the heterogeneous population of patients was enrolled (38). This study favored PCI for the improvement in OS (HR: 0.57; 95% CI: 0.47–0.69; *P* < 0.001) and decrease in the incidence of BM (relative risk = 0.47, 95% CI: 0.33–0.69; *P* < 0.01). In this study, the doses and timing of PCI did not strictly follow unified standards.

Maeng et al. conducted a meta-analysis and systematic review (39) in which they designated OS as the primary endpoint and included primary and secondary analyses of prospective data only. Overall, two of the six articles identified were primary analyses, i.e., the EORTC and JCOG trials. This meta-analysis found no benefit of PCI for OS (HR: 0.82; 95% CI: 0.60–1.11; *P* = 0.19). However, the PCI group showed significantly higher 1-year survival (37.1% vs. 27.1%; HR: 0.87; 95% CI: 0.80–0.95; *P* = 0.002) and PFS (HR: 0.83; 95% CI: 0.70–0.98; *P* = 0.03) and decreased risk of BM (HR: 0.34; 95% CI: 0.23–0.50; *P* < 0.001) than the non-PCI group.

The use of anti-angiogenic tyrosine kinase inhibitors may have been a confounding factor in the CALGB 30,504 study (40), a phase II randomized trial of sunitinib in patients with ES-SCLC who responded to platinum-based chemotherapy, but that study is still worth mentioning. While brain imaging was required before enrollment, it was up to the treating physician to decide whether to perform PCI or not. Notably, patients treated by sunitinib with PCI gained better PFS and OS. The rate of CNS progression was significantly higher in the non-PCI arm than in the PCI arm (27% vs. 12%, *P* = 0.05).

## Toxicity of PCI

Currently, PCI is recommended by many organizations for the treatment of patients with SCLC. However, in recent years, the controversy about the value and indication of PCI remains. With the extension of survival time, the problem of long-term nerve injury after PCI has become increasingly prominent. Many patients reported cognitive and memory disorders half a year after radiotherapy (41). In addition, concerns remain regarding a possible decline in neurocognitive function (NCF) in patients undergoing PCI. Almost all studies on SCLC patients treated with PCI have shown a decrease in the CNS metastases compared with controls. It is assumed that good control of the CNS diseases would lead to a better QoL, but this has not been prospectively proven. Most of the available data have focused on demonstrating that PCI does not result in reduction in NCF and QoL. However, the dose and fraction of PCI varied widely in these trials, as well as the neurocognitive assessment methods did.

A systematic review of the health-related QoL in SCLC patients provided the insight that only the diagnosis of SCLC impaired QoL (42). The study found that patients with good response to treatment had better QoL, indicating that the severity of the disease and response to treatment also affected QoL. Another study suggested that long-term survivors of SCLC may experience significant neurotoxicity due to PCI treatment (43).

Studies of PCI in NSCLC found that although PCI reduced the CNS disease, there were significant cognitive differences in favor of the control arm (41, 44). A randomized phase III trial on using PCI to NSCLC patients (44) pointed out that there were less patients in the PCI arm who developed symptomatic BM, and the time to develop symptomatic BM was much longer than that in the observation arm. Meanwhile, 26 of the 88 patients developed grade 1 and 2 memory impairments, and 16 developed cognitive impairment. The reduction in QoL could be observed in both groups with a median follow-up of 48.5 months. The demographic characteristics (e.g., age and smoking history) of patients enrolled in the study with stage III disease who received chemoradiotherapy were similar to those of patients with LS-SCLC.

PCI has been shown to be associated with increased symptoms in two prominent prospective trials of PCI. The EORTC trial (10, 45) analyzed short-term health-related QoL and patient-reported symptoms and found that from the time of enrollment to 9 months after, there was no significant difference in global health status between the PCI and observation groups (*P* = 0.10). Patients received PCI doses of 20 Gy in 5 or 8 fractions, 24 Gy in 12 fractions, 25 Gy in 10 fractions, and 30 Gy in 10 or 12 fractions, respectively. Although there were no significant differences in role function (*P* = 0.17), cognitive function (*P* = 0.07), and emotional function (*P* = 0.18), the PCI group scored lower for most of the periods in all of these assessments. PCI affected the severity of symptoms of fatigue, loss of appetite, nausea, and leg weakness. The incidence of alopecia and fatigue in the PCI group was significantly higher than that in the control group (*P* < 0.001). Alopecia and fatigue were the most significant side effect of PCI. This suggests that prevention of symptomatic CNS disease may not lead to improvements in QoL or NCF.

The only parameter evaluated in the Japanese trial was the Mini-Mental State Examination (MMSE) (11). In the JCOG trial, PCI was associated with mild acute toxicity, mainly grade 2 toxicity, but there was no difference in the MMSE at 6 and 12 months from baseline. The most common grade 3 adverse events (AEs) observed in the study were anorexia (5% in the PCI group vs. 2% in the observation group), malaise (3% in the PCI group vs. 1% in the observation group), and muscle weakness (1% in the PCI group vs. 5% in the observation group), with similar grade 3 or higher toxicity scores in both groups. There was a lack of assessment of the impact on QoL in this trial. In addition, this experiment suggested that it was necessary to evaluate the effect of chemotherapy on NCF by the result that indicated that majority of patients may have already had abnormal neuropsychological test results before PCI after receiving chemotherapy.

In a randomized trial, the QoL and NCF in patients with locally advanced NSCLC were compared with those in patients receiving PCI. Patients received PCI at a total dose of 30 Gy/15 fractions (41). The study did not find any significant differences in NCF or QoL between the two groups, but there was a significant decline in memory at 1 year. It is worth mentioning that the PCI dose used in this trial was higher than the recommended dose. A review by Tallet et al. (46) analyzed the effects of WBRT either as PCI or as a treatment for BM on NCF in patients with BM. In most studies, the incidence of NCF damage within 1 year of PCI treatment was very low. Another retrospective study reported acute and late AEs in irradiated patients. A meta-analysis by Chen et al. (28) reported that acute toxicity of grade 3 or higher was generally lower in the PCI group (2.2% of grade 3 headache, no grade 4 acute side effects, and no grade 3 or 4 late effects), with the most common grade 2 acute side effects being headache (6.7%) and nausea and vomiting (4.4%).

Given that the increase in neurological side effects could also be related to the age of the patients, PCI was performed less frequently in elder patients, even though there was no age limit in the treatment guidelines (47). This problem is especially prominent in elderly patients with other comorbidities or poor general conditions. Although clinical data of elderly patients support the effectiveness of PCI, it appears to cause a slight increase in AEs (39). The RTOG0212 study indicated that 62% of patients who received PCI (25 Gy, 10 fractions) had long-term neurological responses, among which age of >60 years was the most important risk factor (48, 49). At the same time, elderly patients tend to have poorer performance and more comorbidities that may cast doubt on the need for PCI because of the poor baseline status. However, most current low-level evidence-based results are guiding the treatment of the elderly. Studies have shown that although 67% of lung cancers are diagnosed in older patients who are aged over 65 years, the proportion of elderly patients enrolled in clinical trials is only 35% (50). Therefore, there is no conclusion on whether PCI should be performed in elderly patients. However, in a consensus reached at the European Society for Therapeutic Radiation Oncology and the International Association for the Study of Lung Cancer, experts continued to support that PCI was appropriate to use in elderly patients (51).

It is worth mentioning that the MMSE or Hopkins Language Learning Test-Revised (HVLT-R) was commonly used in various studies to evaluate NCF. The MMSE is by far the most well-known, widely circulated and studied NCF evaluation scale because of its simplicity and ease of administration. However, the MMSE is susceptible to the effects of age, education, cultural background, and even ethnicity. Not only are more educated patients prone to a “ceiling effect,” i.e., possible false negatives, but also less educated people are prone to a “floor effect”, i.e., possible false positives. In contrast, the HVLT-R may be a more reliable assessment method.

Confounding factors, such as the disease status, paraneoplastic syndrome, radiation dose and schedule, concurrent or continuous chemotherapy use, age, and effect of smoking even in the presence of undiagnosed micro-metastases, may influence the neurocognitive assessment after PCI. Patients with underlying depressive symptoms, attentional memory, and problem-solving abilities may also affect the neurocognitive assessment. Patients who already have neurocognitive impairment should be cautious of PCI treatment.

Many of the problems in this field can now be addressed by several developments made to counteract the adverse impact of PCI on NCF. These advances reduce the risk of radiation-related neurocognitive toxicity using new strategies, such as radioprotective pharmacological agents, dose reduction, and hippocampal avoidance (HA) radiotherapy.

## Risk Mitigation

The hippocampus is a structure of the limbic system, which is critical for memory formation. Several studies have shown that NCF is related to the generation of new hippocampal neurons; therefore, protecting this region can reduce NCF damage (52, 53). Several studies have attempted to detect brain structural changes in SCLC patients treated with PCI (54), creating the possibility of reducing the impact of PCI by protecting the hippocampus. Because of the excellent conformability, intensity-modulated radiotherapy (IMRT) can reduce the neurological effects of PCI by protecting the hippocampal neural stem cell compartment during WBRT. Since studies have found a low incidence of BM in the hippocampus, PCI with HA has been shown to be safe for SCLC patients (55). A phase II trial that enrolled 113 patients convincingly demonstrated that avoiding the hippocampal dentate gyrus IMRT during WBRT preserved memory and QoL compared with historical series (56).

Recently, a randomized phase III trial of 150 SCLC patients who received PCI with or without HA was published (57). The results showed HA-PCI could preserve NCF, showing much lower decline in delayed free recall in the HA-PCI group from baseline to 3 months (57). There were no differences between the PCI arm and the HA-PCI arm in OS, QoL, and BM incidence. In addition, a phase III study attempted to use memantine, a drug used to treat moderate-to-severe dementia, in the treatment of BM with WBRT, with or without HA. The results showed that the patient’s symptoms were improved, and the NCF in the HA-PCI group was better protected while the similar intracranial control was gained (58). The NRG CC003 is an ongoing randomized trial of phase II/III PCI for SCLC. The phase II portion of NRG CC003 was designed to compare intracranial relapse rate between the HA-PCI arm and standard PCI arm. This trial will terminate the phase III portion, which was designed to investigate whether HA-PCI reduces cognitive deterioration at 6 months on the Hopkins Language Learning Test-Revised (HVLT-R), if the rate of intracranial recurrence will be higher in the HA-PCI arm. It should be noted that previous smaller randomized trials of HA reported conflicting conclusions about the effects of HA on cognition (57, 59). A prospective study of HA-PCI in patients with LS-SCLC showed that hippocampal protection reduced neuropsychological sequelae associated with brain radiation (60). However, it also suggested that there was a risk of failure in the protected region.

Guo et al. and Kundapur et al. analyzed the incidence of hippocampal metastasis (55, 61). Guo et al. performed a retrospective analysis of the clinical features and patterns of BM in patients with SCLC at diagnosis or during follow-up (61). Meanwhile, Kundapur et al. assessed the risk of hippocampal metastases in patients with SCLC at presentation and after WBRT (55). Both studies found that the incidence of para-hippocampal metastases in SCLC patients may be low enough to be acceptable, suggesting the rationality of the HA technique in SCLC.

Owing to the use of memantine, a drug believed to reduce excitotoxic glutamate release in the brain, the randomized phase III RTOG 0614 trial showed improved neurocognitive function during WBRT combined with memantine administration (62).

Radiotherapy dose and fraction also affect NCF. In a randomized study to explore the optimal radiation dose for PCI, no significant differences were found between the 25 and 36 Gy groups in terms of slight deterioration of communication time, intelligence, and memory deficits (48). Higher doses do not imply higher local control rates and might impair NCF. The doses varied from 20 to 30 Gy in PCI-related studies, resulting in different bioequivalent doses (up to 39 Gy with an α/β of 10 Gy). Finally, the standard dose was established as 25 Gy in 10 fractions.

The development of radiotherapy techniques may reduce the toxicity of PCI. A more reasonable dose and fraction method of PCI can significantly reduce the potential morbidity. The addition of new approaches such as HA and the use of protective agents such as memantine may also enhance the therapeutic potential of PCI (63). However, in the absence of evidence of a significant improvement in survival, the promotion of PCI requires evidence that demonstrates that it is superior in symptoms and QoL than just observed by MRI and received the salvage treatment after disease progression.

## Role of PCI in the Immune Checkpoint Inhibitor Era

Evidence suggests that system therapy is increasingly effective in treating diseases of the CNS. An editorial published in 1995 noted that the beneficiaries of PCI tended to be patients who received less effective chemotherapy regimens, resulting in a higher incidence of BM (64). Standard cytotoxic agents used in the treatment of SCLC are active against the established CNS diseases (65) and with the improvement of systemic treatment, the need for preventive treatment such as PCI will decrease. With ongoing randomized trials in PCI, several studies are being conducted in the ES-SCLC field to improve systemic disease control. Anti-programmed death-1/programmed death ligand-1 agents have recently been shown to be effective in patients with ES-SCLC and are being evaluated in those with LS-SCLC (66). In addition, anti-programmed death-1 therapy was associated with a reduced risk of CNS relapse in the Pacific Phase III NSCLC study (67).

Immunotherapy challenged the role of PCI in ES-SCLC patients, whereas the IMpower133 trial confirmed the survival benefit of atezolizumab in ES-SCLC patients (66). A total of 403 patients were randomized into four-cycle etoposide-combined platinum chemotherapy with atezolizumab or placebo groups, followed by atezolizumab or placebo for treatment maintenance. The results favored the atezolizumab group with a median OS of 12.3 months (95% CI: 10.8–15.9) versus 10.3 months (95% CI: 9.3–11.3) in the placebo group. According to the IMpower133 trial, when the FDA approved immune checkpoint inhibitor (ICI) atezolizumab in combination with etoposide and platinum chemotherapy in 2019, there was an OS benefit for ES-SCLC, which promoted the progress in the systematic treatment of ES-SCLC (66). PCI was not mandatory in the trial protocol: 10% of patients received PCI during the maintenance phase of immunotherapy and did not report the occurrence rates of BM or neurological death. This may reflect concerns about CNS damage caused by PCI and a possible paradigm shift in treatment. Similarly, a phase III randomized multicenter study of nivolumab alone or in combination with ipilimumab, which was conducted in ES-SCLC patients (Checkmate 451), did not mandate PCI but rather determined whether PCI should be administered to patients after first-line chemotherapy based on local standards of care. Another ICI Phase III trial, the CASPIAN Trial, published in 2019 also showed an improvement in OS with the addition of duvalumab (68). The use of PCI in this trial was also poorly controlled, i.e., limited to patients in the chemotherapy group, and only 8% of participants received PCI. Additional studies are needed to evaluate the safety and efficacy of PCI in patients with ES-SCLC undergoing immunotherapy.

The lack of proper controls in these randomized trials limits our understanding of the benefits of introducing ICIs in PCI. Nevertheless, with the addition of ICI, systemic disease control was improved and OS prolonged; therefore, good control of the CNS diseases may become more important. However, because ICIs could penetrate the blood–brain barrier and cause objective responses in patients with known BM, its use may also be sufficient to improve the control of microscopic CNS diseases to replace the benefits of PCI or even WBRT (69).

## Conclusion

At present, studies have shown that craniocerebral MRI can improve the detection rate of subclinical lesions in SCLC, and that PCI can reduce the incidence of BM in patients with ES-SCLC. In addition, regular follow-up MRI and salvage SBRT after BM are the modalities used for SCLC treatment. Although in the past, multiple BM lesions were usually treated with WBRT, Yamamoto et al. showed that patients with more than four intracranial BM lesions treated with SBRT obtained no different OS than those with fewer than four lesions (70, 71). This study suggested the therapeutic potential of stereotactic radiation therapy in multiple BM lesions, while the study by Ruggero et al. confirmed the feasibility of SBRT in the treatment of multiple BM lesions at a technical level (71). This allowed patients to avoid nerve damage from WBRT even if they presented with multiple BM lesions. The SWOG trial will clarify the role of PCI in this field through modern staging and treatment. With advances in the knowledge on the biology of CNS metastasis, high-risk population with metastases can be better identified, and a more reasonable PCI treatment plan can be developed. Owing to modern diagnostic methods and treatment strategies, the clinical value of PCI has been relatively weakened, and the number of patients suitable for PCI has gradually decreased. Therefore, in future research, the clinical value of PCI should be more extensively evaluated, and the indications for PCI should be refined for patient benefit.

## Author Contributions

SX, HZ, SY, QW, and XJ substantially contributed to the conception, drafting, editing, and final approval of this manuscript.

## Conflict of Interest

The authors declare that the research was conducted in the absence of any commercial or financial relationships that could be construed as a potential conflict of interest.

## Publisher’s Note

All claims expressed in this article are solely those of the authors and do not necessarily represent those of their affiliated organizations, or those of the publisher, the editors and the reviewers. Any product that may be evaluated in this article, or claim that may be made by its manufacturer, is not guaranteed or endorsed by the publisher.
